# Quantifying Motor Experience in the Infant Brain: EEG Power, Coherence, and Mu Desynchronization

**DOI:** 10.3389/fpsyg.2016.00216

**Published:** 2016-02-18

**Authors:** Sandy L. Gonzalez, Bethany C. Reeb-Sutherland, Eliza L. Nelson

**Affiliations:** Department of Psychology, Florida International University, MiamiFL, USA

**Keywords:** infants, EEG power, EEG Coherence, mu desynchronization, motor development, cognitive development, social development

## Abstract

The emergence of new motor skills, such as reaching and walking, dramatically changes how infants engage with the world socially and cognitively. Several examples of how motor experience can cascade into cognitive and social development have been documented, yet a significant knowledge gap remains in our understanding of whether these observed behavioral changes are accompanied by underlying neural changes. We propose that electroencephalography (EEG) measures such as power, coherence, and mu desynchronization are optimal tools to quantify motor experience in the infant brain. In this mini-review, we will summarize existing infant research that has separately assessed the relation between motor, cognitive, or social development with coherence, power, or mu desynchronization. We will discuss how the reviewed neural changes seen in seemingly separate developmental domains may be linked based on existing behavioral evidence. We will further propose that power, coherence, and mu desynchronization be used in research exploring the links between motor experience and cognitive and social development.

## Introduction

For infants, learning new motor skills can fundamentally alter their experiences with the world and with others (Campos et al., 2000). Learning a new motor skill may result in a “setting event,” where said motor skill increases the likelihood of producing other actions, resulting in a cascade effect in non-motor domains (Bushnell and Boudreau, 1993; Campos et al., 2000; Clearfield, 2011). Experience with sitting, crawling, walking, and motor-exploratory behavior can have cascading effects in object knowledge (Soska et al., 2010), spatial search (Kermoian and Campos, 1988), language (Walle and Campos, 2014), and academic achievement (Bornstein et al., 2013). Moreover, changes in motor development alter the bidirectional relationship between infants and caregivers, as well as infant attention to social stimuli (Campos et al., 1992; Libertus and Needham, 2011; Karasik et al., 2014). However, research on infant development has yet to decipher the neural mechanisms underlying these documented motor cascades into cognitive and social development. Research on the neural underpinnings of these dynamic interactions across domains would allow for better understanding of infant development, complementing behavioral evidence.

Using electroencephalography (EEG), researchers have begun to elucidate links between motor experience and neural plasticity (Bell and Fox, 1996; Corbetta et al., 2014; Cannon et al., 2015). Crucially, infant motor experience has been linked to neural reorganization (Corbetta et al., 2014). Research implementing EEG measures like power, coherence, and mu desynchronization, in conjunction with motor measures, affords unique information on how motor experience may alter infant neural activity and connectivity. Although these studies are useful, most lack data on how observed motor-neural links relate to existing behavioral literature identifying motor experience as relevant for social and cognitive development. The relation between motor development and other domains has been described as one of “reorganization,” where motor experience can alter an infant’s environment via reorganization of their interactions with social partners and objects (Gustafson, 1984; Biringen et al., 1995; Campos et al., 2000). However, does motor-related *neural reorganization* play a role in subsequent cognitive and social changes? EEG readily provides the tools to answer such questions regarding motor experience in the infant brain.

In this mini-review, we synthesize existing infant EEG literature on motor, cognitive, and social development. We discuss how seemingly isolated EEG findings within each domain may relate to motor development based on behavioral evidence linking motor experience with cognitive and social abilities. First, however, we provide an overview of EEG and optimal EEG measures for quantifying motor experience in the brain: power, coherence and mu desynchronization.

## EEG: Power, Coherence, and Mu Desynchronization

Electroencephalography measures real time electrical activity at the scalp via electrodes in a non-invasive and comfortable manner (Bell and Cuevas, 2012). EEG has great tolerance to movement compared to other neuroimaging methods (i.e., MEG, fMRI). During infancy through early childhood, 6–9 Hz is the most commonly examined EEG frequency band (Marshall et al., 2002; Bell and Cuevas, 2012). Using EEG, researchers can study specific attributes or patterns of electrical activity like power, coherence, or mu desynchronization.

Electroencephalography power reflects the electrical activity of a particular group of neurons, providing a measure of activity within a cortical region as projected to the scalp. Power is calculated as voltage squared within a given frequency band. Because it is measured over time, power can identify sustained area specific patterns of electrical activity at baseline or during a task (Bell, 1998). In infants, increases in power may reflect neural maturation within a cortical area (Bell, 2001). Differences in power between regions can provide information on differential patterns of maturation and activation across the cortex. If power is used in combination with behavioral measures of motor development, links between region specific activity and motor experience can be explored.

Coherence is the squared cross-correlation of activity between spatially distinct electrode sites, providing the degree of interconnection between regions (e.g., strength and number of axons; Nunez, 1981; Thatcher et al., 1987)^1^. Bell and Fox (1996) have previously proposed that increased coherence seen when learning a new skill is likely associated with synaptic growth, indicating integration of function across cortical regions. A subsequent decrease in coherence after greater experience with a skill may be associated with synaptic pruning, indicating greater regional differentiation/specialization and neural efficiency. Importantly, if changes in coherence coincide with changes in motor experience or ability, a strong argument can be made regarding the role of motor experience on neural reorganization and plasticity.

Finally, mu desynchronization is largely a motor experience-dependent electrical pattern measured over the sensorimotor cortex, typically at central electrode sites (Cuevas et al., 2014). When infants observe an individual execute a goal-directed action or when the infant executes an action, mu rhythm becomes desynchronized (i.e., decreased power) compared to mu activity during rest (Marshall et al., 2011). To date, the predominant view suggests that mu desynchronization over central regions reflects mirror neuron activity of an extended fronto-parietal network involved in action coordination and execution (Pineda, 2005; Vanderwert et al., 2013; Thorpe et al., 2015), although recent studies suggest that mu desynchronization may be related to a broader neural network which includes the mirror neuron system (Arnstein et al., 2011; Braadbaart et al., 2013). Critically, mu rhythm (and mirror neurons) may bridge action perception and action production, given that the indexed electrical activity responds most to observed goal-directed actions and that mu desynchronization during action observation is dependent on the infant’s motor repertoire (Pineda, 2005; van Elk et al., 2008; Marshall et al., 2011; Reid et al., 2011). Recently, mu desynchronization was proposed as a possible measure of early social learning (Cannon et al., 2014).

## Use of Power, Coherence, and Mu Desynchronization

In the following sections we review extant EEG literature on infant motor, cognitive and social development implementing power, coherence, and mu desynchronization. Throughout, we discuss how seemingly disconnected EEG findings across all three domains may be linked based on documented behavioral connections between motor development and cognitive and social abilities.

### Motor Development

To our knowledge, Mizuno et al. (1970) and Bell and Fox (1997) have been the only individuals to study motor development and EEG power simultaneously. Although Mizuno et al. (1970) did not explicitly seek to investigate coaction between motor experience and power, their longitudinal study found that infants’ ability to hold up their head, sit, stand and walk was accompanied by increased power measured from the occipital region within the 7.17 to 10.30 frequency. Bell and Fox (1997) investigated individual differences at 8 months in crawling, object permanence, and frontal power within the 6–9 Hz band. Eight-month-olds with 1–4 weeks of crawling experience had significantly greater power at medial frontal, lateral frontal, and parietal regions at baseline, compared to pre-locomotor infants or infants with >4 weeks of crawling (Bell and Fox, 1997; object permanence results discussed in the “Cognitive Development” section). Greater power may indicate increased synchrony or coordination of neural activity within a region, which could reflect increased regional maturation and organization (Nunez, 1981; Bell and Fox, 1992). Increased power in frontal and parietal regions may indicate that onset of crawling is related to a greater need for novice crawlers to recruit these regions for motor planning.

Coherence is an excellent measure of neural changes as infants shift from non-crawling to crawling, and from crawling to walking (Bell and Fox, 1996; Corbetta et al., 2014). Bell and Fox (1996) hypothesized that prior to crawling, infants have an overproduction of synaptic connections, and as crawling experience is gained, “pruning” of unnecessary connections occurs, resulting in an inverted U-shape of coherence. Results found that 8-month-old novice crawlers (1–8 weeks crawling) displayed greater coherence over medial frontal/lateral frontal and medial frontal/occipital regions, compared to same-aged non-crawlers (0 weeks) and experienced crawlers (≥9 weeks). Thus, experience crawling is related to neural reorganization. Recently, Corbetta et al. (2014) explored the relation between walking experience and coherence in 12-month-olds. They also found a motor experience-dependent inverted U-shape of coherence: novice walkers had the highest levels of coherence compared to non-walkers and experienced walkers, specifically between lateral frontal and central electrodes (Corbetta et al., 2014). High coherence in novice walkers and low coherence in experienced walkers may point to synaptic growth as infants begin to walk, and pruning once infants gain experience. Low coherence between lateral frontal and central electrodes may indicate that changes in walking ability relate to greater regional differentiation/specialization between frontal and central regions of the cortex, regions known for their role in motor control (Graziano, 2006), and inhibitory control and working memory (Diamond, 1990).

Mu desynchronization has quickly gained prevalence in investigating motor related neural changes during infancy (Cannon et al., 2014; Cuevas et al., 2014). van Elk et al. (2008) studied mu desynchronization in 14- to 16-month-old crawlers. Infants observed videos of other infants walking or crawling. Mu desynchronization at central electrodes was significantly greater when infants observed crawlers compared to walkers. Recently, Cannon et al. (2015) investigated the relation between motor *ability* and mu desynchronization. Nine-month-olds observed an adult reaching for a toy, and were given the opportunity to reach for the toy themselves. Infant reaching and grasping skill was measured by assessing latency to reach, errors, pre-shaping of the hand, and bimanual reaching. Reach latency was related to mu desynchronization during action observation, with shorter latency correlating with greater desynchronization (Cannon et al., 2015). Marshall et al. (2011) provide additional evidence supporting the role of infant motor experience in mu desynchronization. During observation of an action within their motor repertoire, 14-month-olds displayed mu desynchronization over frontal, central, and parietal regions (Marshall et al., 2011). Findings that mu desynchronization occurs during observation of an action within an infant’s motor repertoire suggests a close link between motor experience and mu rhythm activity.

The results reviewed here on motor experience and neural activity lend strong support to the idea of a dynamic motor–neural interaction. Nonetheless, power, coherence, and mu desynchronization have not been used in studies documenting motor development longitudinally. Moreover, it is unknown how these motor-related neural shifts are implicated in behaviorally observed motor cascades. These gaps in the literature must be addressed, as information pertaining to motor-related neural changes over time may help elucidate the role of motor development as a “setting event” in non-motor domains.

### Cognitive Development

A common measure of infant cognitive development used in conjunction with EEG measures is performance on the A-not-B task, a behavioral measure of working memory and inhibitory control. A-not-B performance has been implicated in individual differences in power. Measuring power and A-not-B performance from 7 to 12 months, Bell and Fox (1992) found that infants who were tolerant of longer delays prior to A-not-B object retrieval at 12 months displayed a significant decrease in baseline frontal power from 7 to 8 months, and had the greatest monthly increase in frontal power from 9 to 10 months, a different neural trajectory from infants tolerating only short delays. When grouping participants by infants who solved the A-not-B task at 7 or 8 months or infants who solved the A-not-B task at 9 months, infants who solved the A-not-B task by 7–8 months displayed greater power at the right frontal lead, compared to the left frontal lead at 8 months (Bell and Fox, 1992). When measuring power during A-not-B engagement, high performing 8-month-olds displayed an increase in power from baseline to task across frontal pole, medial frontal, parietal, and occipital electrodes, while low performers did not show a significant change in power from baseline to task (Bell, 2001). Measuring power at baseline before A-not-B engagement, 8-month-olds who completed the A-not-B task successfully had greater power at medial frontal and occipital electrodes compared to unsuccessful infants (Bell and Fox, 1997). Overall, research on power and the A-not-B task suggests that neural maturity, particularly in frontal regions, is linked to performance on this cognitive task.

Studies measuring coherence during the A-not-B task find that high A-not-B performers at 8 months display significantly lower right hemisphere coherence between frontal pole-medial frontal regions compared to left frontal pole-medial frontal hemisphere coherence, while low performers show no hemispheric differences in coherence between these regions (Bell, 2001). Longitudinally, behavioral differences on A-not-B performance overlap with neural changes: high and low performers on the A-not-B task diverged in performance around 10 months, coinciding with the age when high performers begin to demonstrate an increase in left hemisphere coherence from 10 to 12 months (based on averaged frontal-parietal and frontal-occipital data), evidence of distinct neural trajectories based on cognitive ability (Bell and Fox, 1992). Frontal coherence was also found to increase when greater inhibitory control was needed by 10-month-olds during the A-not-B task (Cuevas et al., 2012). Focusing on A-not-B performance and coherence at 8 months and at 4.5 years, Bell and Wolfe (2007) found changes in coherence, with all electrode pairs demonstrating decreased coherence from baseline to task at 8 months. By 4.5 years of age, increased coherence (during a different measure of working memory) was found only between medial frontal/posterior temporal pairs and medial frontal/occipital pairs (Bell and Wolfe, 2007). Although both Bell (2001) and Bell and Wolfe (2007) measured coherence in samples of 8-month-olds, one study found changes in coherence only between right frontal pole-medial frontal regions, while the other identified changes in coherence across all electrode pairs measured. The differences in coherence seen in Bell (2001) and Bell and Wolfe (2007) samples likely stems from differential participant grouping between the two studies. Although both studies analyzed baseline-to-task changes in EEG, Bell (2001) grouped infants as high and low performers, while Bell and Wolfe (2007) did not group infants based on performance.

Currently, no study has implicated mu desynchronization with cognitive performance. Perhaps researchers conceptualize mu desynchronization as an index of motor experience only, without considering the behavioral links between motor and cognitive development. We speculate that infants who are successful at tasks like A-not-B may display increased mu desynchronization during observation and execution, but actual research is needed to assess how cognition and mu rhythm relate. We know from Smith et al. (1999) and Smith and Thelen (2003) that errors produced by 8- to 10-month-olds on A-not-B tasks disappear by changing the infant’s physical state, like altering posture from sitting to standing between trials. Developmental shifts in posture reorganize infants’ experiences with objects, changing multimodal exploration and subsequent object knowledge (Soska and Adolph, 2014; Soska et al., 2010). Moreover, Kermoian and Campos (1988) found that A-not-B performance is correlated to locomotor experience: infants with experience locomoting voluntarily (crawling or in a walker) perform better on the A-not-B task than pre-locomotor infants. Thus, motor experience plays an important role in cognitive development. To date, only Bell and Fox (1997) have attempted to connect motor development (crawling), neural development (power) and cognitive performance (A-not-B task), but no interaction between all three was identified. Future work should investigate how motor experience is linked to observed neural changes concurrent with performance on cognitive tasks.

### Social Development

Research on power and infant social development has focused on asymmetrical power between hemispheres. Differential power between left and right frontal regions is considered a marker of individual differences in emotional reactivity to stress with withdrawal-related behaviors being related to greater right versus left frontal activation (Davidson and Fox, 1989). Ten-month-olds who cried during maternal separation in Davidson and Fox (1989) displayed greater right frontal activation during baseline measurement. Similarly, infants identified as behaviorally inhibited at 4 months, who continued to be socially inhibited in early childhood, displayed right hemisphere asymmetry at 9, 14, and 48 months (Fox et al., 2001). Negative reactivity in 9-month-olds with greater right frontal asymmetry was associated with social wariness at 4 years (Henderson et al., 2001). Moreover, 9-month-olds who experienced lower quality maternal caregiving behavior were more likely to have right frontal asymmetry at 3 years (Hane et al., 2010). It is important to note that in studies of temperament, classification regarding inhibition is routinely based on infant *motor activity* in response to novel events (Calkins et al., 1996).

Coherence has also served as a neural tool in relation to infant social development, specifically in research on initiating joint attention (IJA; Mundy et al., 2000, 2003). Mundy et al. (2000) found that lower coherence (i.e., greater differentiation/specialization) between left frontal/central sites at 14 months related to greater IJA at 18 months. Left hemisphere coherence between frontal/central sites at 14 months was also negatively correlated with vocabulary at 24 months, and coherence between left frontal/occipital sites was positively correlated with vocabulary (Mundy et al., 2003). Additionally, greater IJA skill at 14 months was highly associated with greater vocabulary at 24 months (Mundy et al., 2003). Thus, individual differences in infants’ IJA and neural development are both related to future vocabulary.

Mu desynchronization has been used as a neural measure in studies of infants’ action perception and imitation (Southgate et al., 2010; Saby et al., 2012). When 8-month-olds were presented with videos of goal-directed versus non-goal-directed actions, mu rhythm over central and right frontal regions decreased during observation of goal-directed actions (Nyström et al., 2011). Moreover, when 9-month-olds were presented with stimuli of mimed reaching actions (i.e., non-goal-directed) and stimuli of a grasping hand disappearing behind an occluder (where grasping can only be inferred), mu desynchronization only occurred during occluded grasping, indicating that infants may predict the goal of a social partner (Southgate et al., 2010). Concerning imitation, Saby et al. (2012) found that 14-month-olds displayed greater mu desynchronization over central regions when their actions were imitated. Overall, evidence suggests that mu desynchronization may be implicated in processing of another individual’s actions, which is critical for social cognition.

From behavioral work, it is known that motor experience plays an important role in social development (Campos et al., 2000). For example, infant IJA mediates the relation between self-locomotion and anticipatory gaze during means-ends sequences, indicating a close link between locomotion, IJA and social cognition (Brandone, 2015). Perhaps the observed relation between IJA, vocabulary and coherence is a function of motor experience, given that language and motor skills are closely interrelated (Nelson et al., 2014; Walle and Campos, 2014). Moreover, paradigms that alter motor experience prior to the typical onset of a motor skill result in social changes, like increased attention to faces during preferential looking, and dishabituation to “unexpected” goals when observing an individual’s goal-directed actions (Sommerville et al., 2005; Libertus and Needham, 2011). Based on the power, coherence, and mu desynchronization literature, it is clear that social development and neural changes interact, but how motor development is implicated in this interaction remains unknown.

## Conclusion

As reviewed here, research utilizing power, coherence, and mu desynchronization provides great insight on motor, cognitive, and social development as *isolated* domains. Research has yet to gain traction on implementing these EEG tools toward understanding the neural mechanisms underlying documented motor cascades in infant cognitive and social development. Development in one domain is likely not isolated from development in other domains, but instead involves dynamic interactions throughout development (Spencer et al., 2011). Much work is left to be done, as little is known regarding the longitudinal development of measures like mu desynchronization, or how power, coherence, and mu desynchronization relate to our existing knowledge of motor, cognitive and social development. Importantly, while existing behavioral work is supportive of motor to cognitive, and motor to social cascades (e.g., Campos et al., 2000; Libertus and Needham, 2011; Walle and Campos, 2014), inclusion of neural measures in research on these cross-domain relations may further clarify the complex interaction between neural plasticity and behavior. One initial step to help clarify these relations would be to conduct more longitudinal work relating motor, cognitive, and social development to neural changes, as most of the research reviewed here was cross-sectional. New research that finds ways to manipulate motor experience in infancy could also provide an optimal way to disentangle how motor experience is related to reorganization of neural activity and connectivity, and how these possible neural changes manifest concurrently across social and cognitive development. Research with EEG comparing typical and atypical development in motor, cognitive, and social abilities is also needed, as it may shed light on how these domains influence each other, and how neural patterns and connectivity play a role in observed behaviors. The plasticity of the brain in infancy lends itself to prime exploration, and we urge researchers to rely on power, coherence and mu desynchronization as tools to explore it.

## Author Contributions

Conceived and wrote the paper: SG, BR-S, and EN.

## Conflict of Interest Statement

The authors declare that the research was conducted in the absence of any commercial or financial relationships that could be construed as a potential conflict of interest.

## Footnotes

1 It is important to note that the measured activity between two electrode sites may also be mediated by a third source (i.e., a common input; Bastos and Schoffelen, 2016).

## References

[B1] Arnstein D. Cui F. Keysers C. Maurits N. M. Gazzola V. (2011). μ-Suppression during action observation and execution correlates with BOLD in dorsal premotor, inferior parietal, and SI cortices. *J. Neurosci.* 31 14243–14249. 10.1523/JNEUROSCI.0963-11.2011 21976509 PMC6623646

[B2] Bastos A. M. Schoffelen J.-M. (2016). A tutorial review of functional connectivity analysis methods and their interpretational pitfalls. *Front. Syst. Neurosci.* 9:175 10.3389/fnsys.2015.00175 PMC4705224 26778976

[B3] Bell M. A. (1998). “The ontogeny of the EEG during infancy and childhood: implications for cognitive development,” in *Neuroimaging in Child Neuropsychiatric Disorders*, ed. Garreau B. (Berlin: Springer-Verlag), 97–111. 10.1007/978-3-642-95848-9

[B4] Bell M. A. (2001). Brain electrical activity associated with cognitive processing during a looking version of the A-not-B task. *Infancy* 2 311–330. 10.1207/S15327078IN0203_2 33451207

[B5] Bell M. A. Cuevas K. (2012). Using EEG to study cognitive development: issues and practices. *J. Cogn. Dev.* 13 281–294. 10.1080/15248372.2012.691143 23144592 PMC3491357

[B6] Bell M. A. Fox N. A. (1992). The relations between frontal brain electrical activity and cognitive development during infancy. *Child Dev.* 63 1142–1163. 10.2307/1131523 1446545

[B7] Bell M. A. Fox N. A. (1996). Crawling experience is related to changes in cortical organization during infancy: evidence from EEG coherence. *Dev. Psychobiol.* 29 551–561. 10.1002/(SICI)1098-2302(199611)29:7<551::AID-DEV1>3.0.CO;2-T 8911771

[B8] Bell M. A. Fox N. A. (1997). Individual differences in object permanence performance at 8 months: locomotor experience and brain electrical activity. *Dev. Psychobiol.* 31 287–297. 9413676

[B9] Bell M. A. Wolfe C. D. (2007). Changes in brain functioning from infancy to early childhood: evidence from EEG power and coherence during working memory tasks. *Dev. Neuropsychol.* 31 21–38. 10.1080/87565640709336885 17305436

[B10] Biringen Z. Emde R. N. Campos J. J. Appelbaum M. I. (1995). Affective reorganization in the infant, the mother, and the dyad: the role of upright locomotion and its timing. *Child Dev.* 66 499–514. 10.2307/1131593 7750380

[B11] Bornstein M. H. Hahn C.-S. Suwalsky J. T. D. (2013). Physically developed and exploratory young infants contribute to their own long-term academic achievement. *Psychol. Sci.* 24 1906–1917. 10.1177/0956797613479974 23964000 PMC4151610

[B12] Braadbaart L. Williams J. H. G. Waiter G. D. (2013). Do mirror neuron areas mediate mu rhythm suppression during imitation and action observation? *Int. J. Psychophysiol.* 89 99–105. 10.1016/j.ijpsycho.2013.05.019 23756148

[B13] Brandone A. C. (2015). Infants’ social and motor experience and the emerging understanding of intentional actions. *Dev. Psychol.* 51 512–523. 10.1037/a0038844 25689000

[B14] Bushnell E. W. Boudreau J. P. (1993). Motor development and the mind: the potential role of motor abilities as a determinant of aspects of perceptual development. *Child Dev.* 64 1005–1021. 10.1111/j.1467-8624.1993.tb04184.x 8404253

[B15] Calkins S. D. Fox N. A. Marshall T. R. (1996). Behavioral and physiological antecedents of inhibited and uninhibited behavior. *Child Dev.* 67 523–540. 10.1111/j.1467-8624.1996.tb01749.x 8625726

[B16] Campos J. J. Anderson D. I. Barbu-Roth M. Hubbard E. M. Hertenstein M. J. Witherington D. (2000). Travel broadens the mind. *Infancy* 1 149–219. 10.1207/S15327078IN0102 32680291

[B17] Campos J. J. Kennoian R. Zumbahlen M. R. (1992). Socioemotional transformations in the family system following infant crawling onset. *New Dir. Child Dev.* 1992 25–40. 10.1002/cd.23219925504 1608513

[B18] Cannon E. N. Simpson E. A. Fox N. A. Vanderwert R. E. Woodward A. L. Ferrari P. F. (2015). Relations between infants’ emerging reach-grasp competence and event-related desynchronization in EEG. *Dev. Sci.* 19 50–62. 10.1111/desc.12295 25754667 PMC7470427

[B19] Cannon E. N. Yoo K. H. Vanderwert R. E. Ferrari P. F. Woodward A. L. Fox N. A. (2014). Action experience, more than observation, influences mu rhythm desynchronization. *PLoS ONE* 9:e92002 10.1371/journal.pone.0092002 PMC3963876 24663967

[B20] Clearfield M. W. (2011). Learning to walk changes infants’ social interactions. *Infant Behav. Dev.* 34 15–25. 10.1016/j.infbeh.2010.04.008 20478619

[B21] Corbetta D. Friedman D. R. Bell M. A. (2014). Brain reorganization as a function of walking experience in 12-month-old infants: implications for the development of manual laterality. *Front. Psychol.* 5:245 10.3389/fpsyg.2014.00245 PMC3968748 24711801

[B22] Cuevas K. Cannon E. N. Yoo K. Fox N. A. (2014). The infant EEG mu rhythm: methodological considerations and best practices. *Dev. Rev.* 34 26–43. 10.1016/j.dr.2013.12.001 24563573 PMC3927793

[B23] Cuevas K. Swingler M. M. Bell M. A. Marcovitch S. Calkins S. D. (2012). Measures of frontal functioning and the emergence of inhibitory control processes at 10 months of age. *Dev. Cogn. Neurosci.* 2 235–243. 10.1016/j.dcn.2012.01.002 22483073 PMC3322365

[B24] Davidson R. J. Fox N. A. (1989). Frontal brain asymmetry predicts infants’ response to maternal separation. *J. Abnorm. Psychol.* 98 127–131. 10.1037/0021-843X.98.2.127 2708653

[B25] Diamond A. (1990). The development and neural bases of memory functions as indexed by the AB and delayed response tasks in human infants and infant monkeys. *Ann. N. Y. Acad. Sci.* 608 267–309; discussion 309–317. 10.1111/j.1749-6632.1990.tb48900.x 2127511

[B26] Fox N. A. Henderson H. A. Rubin K. H. Calkins S. D. Schmidt L. A. (2001). Continuity and discontinuity of behavioral inhibition and exuberance: psychophysiological and behavioral influences across the first four years of life. *Child Dev.* 72 1–21. 10.1111/1467-8624.00262 11280472

[B27] Graziano M. (2006). The organization of behavioral repertoire in motor cortex. *Annu. Rev. Neurosci.* 29 105–134. 10.1146/annurev.neuro.29.051605.112924 16776581

[B28] Gustafson G. E. (1984). Effects of the ability to locomote on infants’ social and exploratory behaviors: an experimental study. *Dev. Psychol.* 20 397–405. 10.1037//0012-1649.20.3.397

[B29] Hane A. A. Henderson H. A. Reeb-Sutherland B. C. Fox N. A. (2010). Ordinary variations in human maternal caregiving in infancy and biobehavioral development in early childhood: a follow-up study. *Dev. Psychobiol.* 52 558–567. 10.1002/dev.20461 20806328 PMC4822519

[B30] Henderson H. A. Fox N. A. Rubin K. H. (2001). Temperamental contributions to social behavior: the moderating roles of frontal EEG asymmetry and gender. *J. Am. Acad. Child Adolesc. Psychiatry* 40 68–74. 10.1097/00004583-200101000-00018 11195566

[B31] Karasik L. B. Tamis-Lemonda C. S. Adolph K. E. (2014). Crawling and walking infants elicit different verbal responses from mothers. *Dev. Sci.* 3 1–8. 10.1111/desc.12129 PMC3997624 24314018

[B32] Kermoian R. Campos J. J. (1988). Locomotor experience: a facilitator of spatial cognitive development. *Child Dev.* 59 908–917. 10.2307/1130258 3168629

[B33] Libertus K. Needham A. (2011). Reaching experience increases face preference in 3-month-old infants. *Dev. Sci.* 14 1355–1364. 10.1111/j.1467-7687.2011.01084.x 22010895 PMC3888836

[B34] Marshall P. J. Bar-Haim Y. Fox N. A. (2002). Development of the EEG from 5 months to 4 years of age. *Clin. Neurophysiol.* 113 1199–1208. 10.1016/S1388-2457(02)00163-3 12139998

[B35] Marshall P. J. Young T. Meltzoff A. N. (2011). Neural correlates of action observation and execution in 14-month-old infants: an event-related EEG desynchronization study. *Dev. Sci.* 14 474–480. 10.1111/j.1467-7687.2010.00991.x 21477187 PMC3106425

[B36] Mizuno T. Yamauchi N. Watanabe A. Komatsushiro M. Takagi T. Iinuma K. (1970). Maturation patterns of EEG basic waves of healthy infants under twelve-months of age. *Tohoku J. Exp. Med.* 102 91–98. 10.1620/tjem.102.91 5495067

[B37] Mundy P. Card J. Fox N. (2000). EEG correlates of the development of infant joint attention skills. *Dev. Psychobiol.* 36 325–338. 10.1002/(SICI)1098-2302(200005)36:4<325::AID-DEV7>3.0.CO;2-F 10797253

[B38] Mundy P. Fox N. Card J. (2003). EEG coherence, joint attention and language development in the second year. *Dev. Sci.* 6 48–54. 10.1111/1467-7687.00253

[B39] Nelson E. L. Campbell J. M. Michel G. F. (2014). Early handedness in infancy predicts language ability in toddlers. *Dev. Psychol.* 50 809–814. 10.1037/a0033803 23855258 PMC4059533

[B40] Nunez P. L. (1981). *Electric Fields of the Brain: The Neurophysics of EEG*. New York, NY: Oxford University Press.

[B41] Nyström P. Ljunghammar T. Rosander K. Von Hofsten C. (2011). Using mu rhythm desynchronization to measure mirror neuron activity in infants. *Dev. Sci.* 14 327–335. 10.1111/j.1467-7687.2010.00979.x 22213903

[B42] Pineda J. A. (2005). The functional significance of mu rhythms: translating “seeing” and “hearing” into “doing.” *Brain Res. Brain Res. Rev.* 50 57–68. 10.1016/j.brainresrev.2005.04.005 15925412

[B43] Reid V. M. Striano T. Iacoboni M. (2011). Neural correlates of dyadic interaction during infancy. *Dev. Cogn. Neurosci.* 1 124–130. 10.1016/j.dcn.2011.01.001 22436436 PMC6987537

[B44] Saby J. N. Marshall P. J. Meltzoff A. N. (2012). Neural correlates of being imitated: an EEG study in preverbal infants. *Soc. Neurosci.* 7 650–661. 10.1080/17470919.2012.691429 22646701 PMC3434237

[B45] Smith L. B. Thelen E. (2003). Development as a dynamic system. *Trends Cogn. Sci.* 7 343–348. 10.1016/S1364-6613(03)00156-6 12907229

[B46] Smith L. B. Thelen E. Titzer R. McLin D. (1999). Knowing in the context of acting: the task dynamics of the A-not-B error. *Psychol. Rev.* 106 235–260. 10.1037/0033-295X.106.2.235 10378013

[B47] Sommerville J. A. Woodward A. L. Needham A. (2005). Action experience alters 3-month-old infants’ perception of others’ actions. *Cognition* 96 1–11. 10.1016/j.cognition.2004.07.004 15833301 PMC3908452

[B48] Soska K. C. Adolph K. E. (2014). Postural position constrains multimodal object exploration in infants. *Infancy* 19 138–161. 10.1111/infa.12039 24639621 PMC3951720

[B49] Soska K. C. Adolph K. E. Johnson S. P. (2010). Systems in development: motor skill acquisition facilitates three-dimensional object completion. *Dev. Psychol.* 46 129–138. 10.1037/a0014618 20053012 PMC2805173

[B50] Southgate V. Johnson M. H. Karoui I. E. Csibra G. (2010). Motor system activation reveals infants’ on-line prediction of others’ goals. *Psychol. Sci.* 21 355–359. 10.1177/0956797610362058 20424068

[B51] Spencer J. P. Perone S. Buss A. T. (2011). Twenty years and going strong: a dynamic systems revolution in motor and cognitive development. *Child Dev. Perspect.* 5 260–266. 10.1111/j.1750-8606.2011.00194.x 22125575 PMC3224018

[B52] Thatcher R. W. Walker R. A. Giudice S. (1987). Human cerebral hemispheres develop at different rates and ages. *Science* 236 1110–1113. 10.1126/science.3576224 3576224

[B53] Thorpe S. G. Cannon E. N. Fox N. A. (2015). Spectral and source structural development of mu and alpha rhythms from infancy through adulthood. *Clin. Neurophysiol.* 127 254–269. 10.1016/j.clinph.2015.03.004 25910852 PMC4818120

[B54] van Elk M. van Schie H. T. Hunnius S. Vesper C. Bekkering H. (2008). You’ll never crawl alone: neurophysiological evidence for experience-dependent motor resonance in infancy. *Neuroimage* 43 808–814. 10.1016/j.neuroimage.2008.07.057 18760368

[B55] Vanderwert R. E. Fox N. A. Ferrari P. F. (2013). The mirror mechanism and mu rhythm in social development. *Neurosci. Lett.* 540 15–20. 10.1016/j.neulet.2012.10.006 23063953 PMC3612380

[B56] Walle E. Campos J. J. (2014). Infant language development is related to the acquisition of walking. *Dev. Psychol.* 50 336–348. 10.1037/a0033238 23750505

